# Umbilical Artery Thrombosis Causing Fetal Distress: A Case Report

**DOI:** 10.7759/cureus.64624

**Published:** 2024-07-15

**Authors:** Jia Li, Iqra Ijaz, Liang Zhao

**Affiliations:** 1 Department of Gynecology, Chongqing Traditional Chinese Medicine Hospital, Chongqing, CHN; 2 Department of Obstetrics and Gynecology, Holy Family Hospital, Rawalpindi, Rawalpindi, PAK; 3 Sichuan Provincial Center for Gynecological and Breast Diseases, Southwest Medical University, Luzhou, CHN

**Keywords:** hypocoiled umbilical cord, clinical analysis, fetal distress, pregnancy complications, nuchal cord, umbilical artery thrombosis

## Abstract

The umbilical cord (UC) is vital to maintain blood circulation between the mother and the growing fetus, which is sometimes disrupted. The umbilical artery thrombosis (UAT) is an infrequent complication of pregnancy that can lead to extreme perinatal outcomes, ranging from intrauterine growth restriction stillbirth to neonatal death. The prenatal diagnosis of UAT is essential and sometimes challenging to detect in clinical practice. Once it is detected, the emergent delivery through a cesarean section is considered after the steroidal lung maturity of the fetus. We report a primigravida diagnosed with this rare pregnancy complication, the UAT at delivery, along with the nuchal cord and abnormally coiled UC. The patient had an uneventful course of pregnancy except for the premature rupture of membranes and continuous fetal distress in the second stage of labor. As the labor progression was optimal, and prioritizing the patient’s desire, she was vigilantly observed under the premise of continuous electronic fetal monitoring (EFM) to facilitate any emergency, ultimately resulting in the spontaneous vaginal delivery of an alive and healthy baby boy. The fetal distress detected through EFM is an indicator of several stressors predisposing the fetus to some unknown danger that carries an increased risk of perinatal mortality. Based on our experience, it is suggested that radiologists should routinely conduct UC sonographic studies on regular antenatal scans; obstetricians should also have a brief and precise awareness of the critical lifesaving sonographic parameters to measure. The UAT, nuchal cord, and abnormal UC coiling, as found in our case, are all rare factors and related to some extent of fetal morbidity and mortality; once such complications are prenatally suspected, one should manage it through close monitoring and timely decision of appropriate delivery time.

## Introduction

The human umbilical cord (UC) from one end is connected to the placenta fetal membranes, and the other is connected to the umbilicus on the fetal abdominal wall. It is a vital life bridge between the mother and the growing fetus. The UC length ranges from 30 to 100 cm long with an average length of 50-60 cm, and the amniotic membrane covers its surface. Three blood vessels, two arteries, and one vein are enclosed in the protective Wharton’s jelly, making up the UC [1].

The umbilical artery thrombosis (UAT) is a condition whereby one out of two umbilical arteries gets blocked at any stage of pregnancy, leaving one artery to take charge of fetal circulation. It is a life-threatening condition for the growing fetus; however, it is relatively rare in clinical practice, with an incidence rate of approximately 0.4% in high-risk pregnancies, 0.08% in delivered newborns, and approximately 0.1% in postnatal autopsies and a higher incidence in male fetuses [2].

The definitive cause of UAT is still unclear, but several factors are considered to play a role in this complication's occurrence, including UC anatomical abnormalities and maternal and fetal factors. The adverse pregnancy outcome with UAT includes intrauterine growth restriction (IUGR), stillbirth, and neonatal death. When performed by experts, the Doppler flow ultrasonography (USG) can make a difference in antenatal UAT diagnosis, and accordingly, management efforts could be sought for a better prognosis. Herein, we report a case of fetal distress in labor where UAT in a singleton-term pregnancy was diagnosed at the delivery of the neonate, along with the nuchal cord and abnormally coiled UC noted at the delivery.

## Case presentation

A 24-year-old primigravid lady was admitted to the obstetrics emergency department of a tertiary care hospital in China. She presented at 03:00 with the complaint of a gush of clear fluid from her vagina an hour ago. On admission, the premature rupture of membranes (PROM) was diagnosed at 39 weeks of gestation from the last menstrual period (LMP). The clinical assessment at admission revealed typical vital signs on physical examination. On obstetric palpation of the uterus, the fundal height was 35 cm with an abdominal circumference of 93 cm, the fetal lie was longitudinal with a cephalic presentation, the estimated fetal weight was about 3000 g, and no uterine contractions were palpable; however, the fetal head was engaged.

On vaginal examination, the cervix was noted to be soft, central with 80% effacement but without any significant dilatation, and the presenting part was at the -2 station. The ruptured fetal membranes were confirmed as the clear amniotic fluid was draining. At admission, the non-stress test (NST) by electronic fetal monitoring (EFM)/cardiotocography (CTG) was reported to be normal of reactive type.

The patient had regular antenatal checkups during pregnancy and found no particular abnormalities. The patient’s white cell count was 5.41 x 109/l, platelet count was 168 x 109/l, and hemoglobin was 108 g/l. The patient reported seronegative for hepatitis B and C and human immunodeficiency virus. Any concerning history related to other medical or surgical issues and family history was unremarkable.

**Figure 1:**
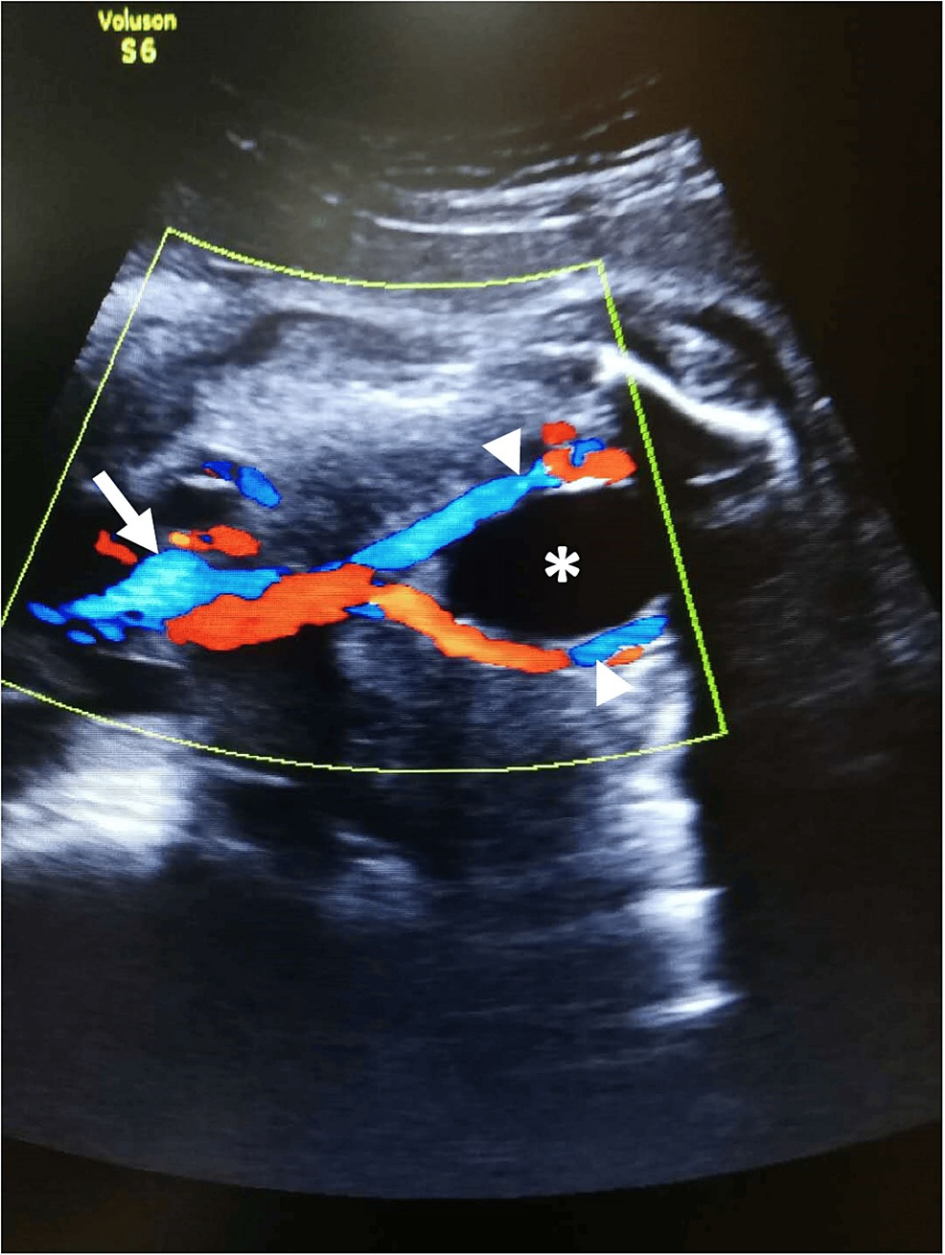
Ultrasound scan with Doppler study The routine ultrasound scan cross-sectional views of ultrasound images at the bladder level (asterisk) showed one umbilical vein (arrow) and two umbilical arteries (arrowhead).

Obstetrics USG during early pregnancy at 23 and later at 35 weeks of gestation showed normal fetal parameters, UC composition, and normal Doppler flow studies (Figure 1).

At admission to the emergency department, the fetal parameters were of term gestation on USG; however, no Doppler flow studies were carried out at that time. Consequently, the patient's labor started at 07:00 after admission. The vaginal examination at 09:30 showed that the cervix was 3 cm dilated with presenting part at the -1 station, and the amniotic fluid was clear. The CTG monitoring showed regular uterine contractions; however, on EFM, the baseline variation of the fetal heart rate was not good, but it was without any evident deceleration.

As the patient had a strong desire to have a vaginal trial of labor, the labor process was observed vigilantly under the premise of continued fetal surveillance with EFM.

The labor progressed quickly, and at 11:30, the cervix was fully dilated, and the patient started the effort to push. The CTG monitoring showed that the baseline variation of the fetal heart rate was becoming poor and early decelerations were occurring frequently; however, the recovery was noticed quickly after contractions. 

The patient successfully delivered a live baby boy with a nuchal cord at 12:15, weighing 3100 g; the amniotic fluid was clear, and the newborn Apgar score was 3 and 10 at one and five minutes, respectively. The newborn's breathing pattern was regular, and the heart sounds were strong and rhythmic. The umbilical vein blood pH value was 7.26.

**Figure 2:**
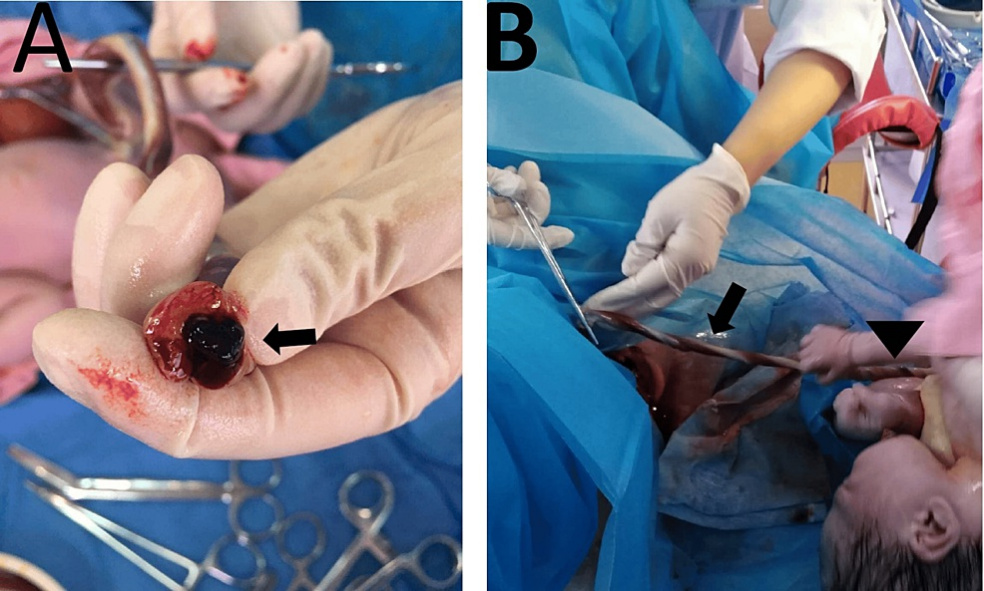
Gross appearance of the umbilical cord Gross appearance of the umbilical cord demonstrating the presence of thrombosis in the umbilical vessel (black arrow) (A); the borderline abnormal umbilical cord coiling/twisting was thin and had a hypocoiled appearance (black arrow), hypercoiled the near fetal abdominal wall (arrowhead) (B).

The UC measured about 50 cm long, with red infiltration of Wharton's jelly outside one umbilical artery, and the other umbilical artery was stiff in texture and appeared dark red with a loss of protecting jelly around it (Figure 2A, 2B).

The cross-section of the UC showed two umbilical arteries; however, one was completely blocked due to old clotted blood and the obsolete thrombus.

**Figure 3:**
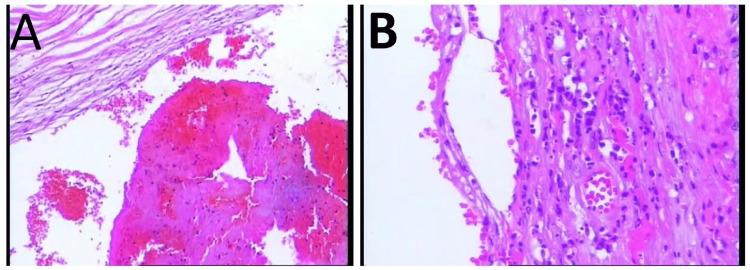
Microscopic examination Microscopic examination: hematoxylin and eosin ×25 magnification, fibrinoid necrosis in the arterial wall, and the thrombosis was found in the umbilical artery lumen (A). Small calcifications were seen on the placental tissue and, and a small amount of acute and chronic inflammation infiltration was seen on the fetal membranes.

The UC, placenta, and fetal membranes were sent for pathologic examination, which showed mature placental tissue, small calcifications, a small amount of acute and chronic inflammatory cell infiltration in the fetal membranes, and a thrombus in an umbilical artery. The pathological examination confirmed the UAT (Figure 3A, 3B).

The child was followed up for two years. The child's growth and development were average, and his intelligence was consistent with those of the same age.

## Discussion

Umbilical vascular thrombosis is a rare complication of pregnancy; however, it is associated with high fetal and neonatal morbidity and mortality, posing the fetus to several adverse pregnancy outcomes [3]. The UAT can be divided into occlusive thrombosis and non-occlusive thrombosis. Occlusive thrombosis has a more profound impact on the fetus; it may lead to fetal growth restriction, severe IUGR, fetal intolerance to the labor process, fetal distress, neonatal cerebral palsy, cerebral hemorrhage, peritoneal encephalopathy, neurological dysfunction, neonatal liver disease, severe lack of protein S after birth, and even intrauterine fetal death; neonatal death and neonates are more susceptible to develop fetal thrombotic blood vessels [3]. UAT is rare in clinical practice, but it is extremely harmful.

The exact cause and the specific course of UAT is not known. However, according to Virchow’s hypothesis, the predisposing factors for thrombi formation, such as endothelial damage, hypercoagulable states, and blood flow stasis-related disease, could provoke UAT [4]. Moreover, thrombosis can occur secondary to abnormalities in the anatomy of the UC, such as the UC being too short, too thin, or too long, the UC torsion, and abnormal connection sites on the placental disc [5]. It may also be related to mechanical damage to the UC, such as excessive twisting, knotting, and compression [5]. Furthermore, fetal coagulation abnormalities, infections, maternal blood pressure, and blood sugar abnormalities, and smoking can predispose the fetus to this life-threatening condition [6,7].

In the present case, the woman had no history of smoking or any other comorbidities, her prenatal examination was unremarkable, and there were no abnormalities seen in the examination after admission. However, the postpartum examination of the placental tissue and fetal membranes showed small calcifications in the placenta along with acute and chronic inflammatory cell infiltration in the fetal membranes, which may be related to the infection.

In addition, the UC at delivery was grossly seen with an abnormal coiling index. Near the fetal abdominal wall, it was borderline hypercoiled, and the remaining UC showed hypocoiling with loss of protecting Wharton’s jelly. It does not rule out that Wharton’s jelly around the UC cannot fully protect the internal umbilical blood vessels, causing them to be easily compressed, possibly leading to thrombosis. A meta-analysis by Pergialiotis et al. used the UC coiling index to predict relevant pregnancy outcomes. It revealed that hypocoiled UC was linked to higher rates of preterm birth, fetal distress, fetal heart rate abnormalities, low Apgar scores of <7 at five minutes, meconium-stained liquor, small for gestational-age neonates, fetal anomalies, neonatal intensive care unit (NICU) admission, and fetal death [8,9].

Furthermore, a recent study by Nikhila et al., which evaluated the UC coiling index in relation to adverse pregnancy outcomes, found no significant effect of the abnormal coiling index in predicting poor perinatal outcomes. Still, they confirmed a strong association between abnormal coiling and maternal anemia and hypertension in pregnancy [10]. As these results were obtained from a small number of patients, a more extensive population-based study needs to be conducted to determine cutoffs for hypo- and hypercoiling and to look for any strong correlation between improper coiling and perinatal outcome.

In this case, the number of umbilical blood vessels was normal on USG at 23 and 35 weeks of gestation. The USG done at 38+2 weeks of gestation showed normal growth parameters and biophysical profile; however, the umbilical blood vessel composition abnormality could not be determined due to obstruction caused by limbs position, and as the EFM was of the typical normal type, the patient was dealt with as a usual case.

At admission to the emergency department, the patient presented with PROM without any complaints of reduced fetal movements or any other alarming signs and symptoms at the beginning, so the patient was observed for smooth progression of labor. The patient developed continuous fetal distress in the second stage of labor; during this critical phase, which was progressing well, she was observed vigilantly under the premise of constant EFM surveillance to achieve a good outcome, and ultimately, the mother successfully delivered vaginally. On delivery, the nuchal cord, UAT, and borderline abnormal coiling of UC were noted.

When the UC is wound around the fetal neck, it is referred to as the nuchal cord; this complication of pregnancy increases relative to an increase in gestational age with an incidence of up to 29%. It can cause impedance in fetal circulation, leading to fetal distress and acidosis; as soon as it is discovered, measures should be taken for expedient reduction to restore blood circulation and avoid fetal asphyxia [11].

On the other hand, the distinction between UAT and single umbilical artery (SUA) mainly relies on ultrasound. The ultrasound manifestations of UAT are that the number of fetal umbilical vessels is normal in the first and second trimesters, but in the third trimester, the blood flow signal of the umbilical artery on one side of the fetal bladder cannot be detected; the inner diameter of one umbilical artery in the free segment of the UC becomes thinner, there is no blood flow signal, and the other umbilical artery has standard blood flow signal [12].

The “grab the orange sign” of umbilical vessel embolism is a reproducible and novel sign that can be used to differentiate between primary dysplasia and secondary embolic obstruction; it is produced secondary to an obstructed artery parallel to the other normal artery that is surrounded by the umbilical vein [5]. The SUA means that there is only one umbilical artery in the UC routine ultrasound throughout pregnancy, which shows that the cross-section of the free section of the UC is "L" shaped, often combined with fetal chromosomal abnormalities and congenital structural malformations.

There are two theories for its occurrence. One theory is that the initial development of the embryo is normal; during the development process, an umbilical artery shrinks and then disappears, and the other theory is that it is congenitally underdeveloped; that is, it develops from the beginning of the embryo, an umbilical artery and an umbilical vein [12]. 

In our case, no structural abnormalities were observed when the conventional color Doppler USG was performed at 23 weeks, and fetal echocardiography was also normal. According to the USG results later in pregnancy, it was considered that the patient had atresia of one umbilical artery in the third trimester of pregnancy. Functional compensation of the other umbilical artery allowed the fetus to adapt to labor. It ultimately resulted in a smooth vaginal delivery with a good pregnancy outcome, which is extremely rare in the clinical practice.

During clinical prenatal checkups, when obstetricians or radiologists discover abnormalities in umbilical vessels, they should carefully inquire about the medical history, understand the underlying diseases of the pregnant woman, compare previous ultrasound examinations during pregnancy, and determine whether SUA or umbilical vessel thrombosis is present before delivery to obtain a good pregnancy outcome. They should conduct careful postpartum examinations to identify the condition of the placenta and umbilical cord.

The literature reports indicate that once UAT is detected, a timely decision for delivery by cesarean section is recommended to terminate the pregnancy to reduce the adverse effects of this morbidity. However, even if the pregnancy is terminated in time, the newborn may still have serious complications [3,5]. The prenatal diagnosis can allow for some time to enable fetal lung maturity with steroidal cover for a better prognosis. Therefore, regular antenatal checkups should be ensured with the provision of optimum USG by experts to detect UC abnormalities, as these can lead to fetal mortality at any stage. Once UAT or other life-threatening conditions related to UC are detected, timely decisions should be made accordingly for a better prognosis under strict fetal surveillance.

Moreover, this life-maintaining cord between mother and fetus postnatally provides intravenous access through umbilical vein catheterization for fetal resuscitation and transfusions for up to 14 days [13]. Moreover, the UC blood is the source of allogeneic hematopoietic stem cells; it has been used in search of a cure for hematopoietic diseases and bone marrow transplantation [14]. The components of the UC, including stem cells and Wharton’s jelly, are under investigation by researchers to look more into their role in disease therapy [15,16].

## Conclusions

The umbilical arterial thrombosis in pregnancy occurs infrequently but carries serious perinatal complications. It puts the fetus more at risk when not detected early on antenatal scans; therefore, it should be taken into account when fetal distress is detected on electronic fetal monitoring. As umbilical cord abnormalities are an independent risk factor for the UAT, it should be routinely practiced to structurally assess the UC on antenatal scans, especially in cases where IUGR is suspected. Although the exact cause of UAT is still a question, obstetricians should practice vigilantly in cases where patients present with predisposing risk factors and timely decisions regarding mode of delivery should be considered for a good outcome. As in the present case, UAT was diagnosed after delivery in a newborn with fetal distress during the labor process, along with abnormal UC coiling and nuchal cord, independent risk factors for UAT. The spontaneous vaginal delivery with UAT is sporadic, as in the present case; however, when on the verge, under strict fetal surveillance and patient’s consent, it may be considered.
